# Prioritizing Support Offered to Caregivers by Examining the Status Quo and Opportunities for Enhancement When Using Web-Based Self-reported Health Questionnaires: Descriptive Qualitative Study

**DOI:** 10.2196/30877

**Published:** 2022-04-08

**Authors:** Theresa Coles, Nicole Lucas, Erin Daniell, Caitlin Sullivan, Ke Wang, Jennifer M Olsen, Megan Shepherd-Banigan

**Affiliations:** 1 Department of Population Health Sciences Duke University Durham, NC United States; 2 Rosalynn Carter Institute for Caregivers Americus, GA United States; 3 Duke-Margolis Center for Health Policy Duke University Durham, NC United States; 4 Center of Innovation to Accelerate Discovery and Practice Transformation Durham Veterans Affairs Health Care System Durham, NC United States

**Keywords:** caregiver, web-based questionnaires, self-report questionnaires, caregiver outcomes, intervention technology, patient-reported outcome measures

## Abstract

**Background:**

The Rosalynn Carter Institute for Caregivers (RCI) offers evidence-based interventions to promote caregivers’ health and well-being. Trained coaches regularly meet with caregivers to offer education and instructions to improve caregiver health, build skill sets, and increase resilience. Two of these interventions, RCI Resources for Enhancing Alzheimer's Caregiver Health (REACH) and Operation Family Caregiver (OFC), use a set of caregiver-reported questionnaires to monitor caregivers’ health status and needs.

**Objective:**

This study aims to describe how web-based assessment questionnaires are used to identify and monitor caregiver status in the RCI REACH and OFC programs and outlines perceived enhancements to the web-based system that could support caregiver-coach encounters by directing priorities.

**Methods:**

This was a descriptive, qualitative study. Data were collected via semistructured interviews with caregivers and coaches in the RCI REACH and OFC programs from July 2020 to October 2020. During the interviews, participants were asked to describe how the assessment questionnaires were used to inform caregiver-coach encounters, perceived usefulness of enhancements to web-based display, and preference for the structure of score results. The interviews were recorded, transcribed, and coded using structural and interpretive codes from a structured codebook. Qualitative content analysis was used to identify themes and summarize the results.

**Results:**

A total of 25 caregivers (RCI REACH: 13/25, 52%; OFC: 12/25, 48%) and 11 coaches (RCI REACH: 5/11, 45%; OFC: 6/11, 55%) were interviewed. Most caregivers indicated that the assessment questions were relevant to their caregiving experience. Some caregivers and coaches indicated that they thought the assessment should be administered multiple times throughout the program to evaluate the caregiver progress. Overall, caregivers did not want their scores to be compared with those of other caregivers, and there was heterogeneity in how caregivers preferred to view their results at the question or topic level. Coaches were uncertain as to which and how much of the results from the self-reported questionnaires should be shared with caregivers. Overall, the results were very similar, regardless of program affiliation (RCI REACH vs OFC).

**Conclusions:**

Web-based and procedural enhancements were identified to enrich caregiver-coach encounters. New and enhanced strategies for using web-based assessment questionnaires to direct priorities in the caregiver-coach encounters included integrating figures showing caregiver progress at the individual caregiver level, ability to toggle results through different figures focused on individual versus aggregate results, and support for interpreting scores. The results of this qualitative study will drive the next steps for RCI’s web-based platform and expand on current standards for administering self-reported questionnaires in clinical practice settings.

## Introduction

### Background

One in five adults in the United States provides unpaid caregiving; these adults are also known as family caregivers [1]. Responsibilities for unpaid caregivers are wide-ranging and include interacting with clinicians on behalf of the care recipient, transportation, housework, shopping, paperwork, managing finances, helping with personal hygiene, and administering medical therapies [2]. Caregiving responsibilities often accumulate over time as the care recipients’ health status worsens, and caregivers must manage the changing needs of their care recipients. For family caregivers, caregiving is associated with higher levels of depression and anxiety, worse self-reported physical health, and increased risk of early death [3].

The Rosalynn Carter Institute for Caregivers (RCI) offers evidence-based interventions to promote the health and well-being of caregivers and provides training on coping skills [4]. RCI coaches regularly meet with caregivers to offer education and training to improve their health, build skill sets, and increase resilience. During these one-on-one meetings, caregivers and coaches touched on a variety of topics, including the burden of caregiving, self-care techniques, problem solving, and community support. Two of these interventions, RCI Resources for Enhancing Alzheimer's Caregiver Health (REACH) and Operation Family Caregiver (OFC), use a set of caregiver-reported questionnaires to monitor caregivers’ health status and needs.

RCI REACH is a coach-led one-on-one program for caregivers that focuses on building caregiver skills in stress and mood management, as well as problem solving. Caregivers in this program care for individuals with Alzheimer's disease and related dementia. The RCI REACH includes 12 in-person or web-based sessions with a coach. On average, it takes approximately 7.5 months for caregivers to complete the program depending on the spacing of the sessions. A preliminary single-group observational study showed that RCI REACH participants had a decrease in depression and caregiver burden, and caregivers reported being less troubled by care-recipient behavioral problems [5].

The OFC is a coach-led one-on-one program for caregivers of active-duty military personnel or veterans. Sessions were conducted in person or via a web-based video platform including FaceTime (Apple Inc) or Zoom (Zoom Video Communications) over the course of 8 sessions (average time to complete the sessions was 5.4 months). Coaches help caregivers recognize challenges, identify solutions, and develop problem-solving skills. Using a single-group, pre-post study design, Easom et al [6] showed that caregivers who participated in the OFC had decreased depression and feelings of caregiver burden, as well as increased life satisfaction and positive problem-solving skills.

Included in each of these RCI programs are a set of web-based questionnaires that are used to provide RCI with information on caregiver outcomes, otherwise known as *the assessment*, which consists of a variety of validated questionnaires, such as the Center for Epidemiologic Studies Depression Scale—Revised [7] to measure depression and the Zarit Burden Interview [8] to evaluate caregiver burden. Other questionnaires validated for caregivers were used to evaluate caregiver work performance [9], work-family conflict [10], self-efficacy for caregiving [11], problem-solving ability [12], life balance [13], caregiver perceived financial stress [14], and community support [15]. As the challenges and needs of caregivers in RCI REACH and OFC are different, the assessment also asks caregivers to report concepts specific to each program. For example, caregivers in the RCI REACH program are asked specific questions about caring for an individual with dementia, including care recipients’ memory and behaviors [16], how bothered they are by their care recipient’s memory status or behaviors, home preparedness and safety, and desire to place the care recipient in a nursing home or assisted living facility [17]. For the OFC, the assessment inquired about problem solving [12] and child anxiety for children living in the caregiver’s household [18].

The web-based assessment is currently administered when caregivers enroll in the program (T1) and when they finish it (T2). Historically, this assessment was primarily used to evaluate the impact of the programs on caregiver outcomes at the aggregate level. However, the RCI was interested in learning what opportunities there might be to expand the usefulness of the assessments and results to coaches and caregivers.

Coach-caregiver encounters are similar to clinician-patient encounters in routine clinical care. Self-reported questionnaires, often referred to as patient-reported outcome (PRO) measures, are administered in clinical care to obtain information on patient status. Significant evidence supports the integration of PROs in clinical care with processes that report scores to patients and clinicians. PROs are associated with improved patient quality of life outcomes, patient-clinician communication, patient satisfaction, and facilitation of meaningful and focused conversations between patients and clinicians [19-30]. The rationale for this study is that, by exploring an analogous process in caregiver-coach encounters, RCI’s web-based assessment could provide real-time feedback about progress on intervention targets and can serve as an intervention strategy to more directly tailor the intervention to improve caregiver-coach communication, support the coach in identifying and prioritizing caregiver needs, and achieve better intervention-related outcomes for caregivers.

### Objectives

We conducted a qualitative study to describe caregiver and coach perspectives and proposed enhancements to the web-based assessment that could support caregiver-coach encounters. Specifically, we were interested in how the assessment would optimally integrate into the coach-caregiver encounter, including how often outcomes should be evaluated, which format of outcome presentation would be most helpful and to whom, how stakeholders would like to see outcomes presented (eg, figures and text), and what specific features of the assessment would support the encounter and shared decision-making about caregiver needs or successes. This project provides foundational knowledge for designing a web-based system that could support caregiver-coach encounters by providing timely feedback on caregiver needs.

## Methods

We conducted a qualitative descriptive study including 1-hour one-on-one semistructured interviews with caregivers and coaches associated with RCI’s REACH and OFC programs from July 2020 to October 2020.

### Data Collection

Semistructured interview guides were developed to standardize the topics discussed during the interviews. The caregiver and coach interview guides were developed separately because of the different perspectives shared by each type of participant. Interview guides were iteratively developed before data collection began and included open-ended questions and scripted probes. During data collection, the team refined the interview guides by modifying the way questions were worded or adding probes to improve the flow of the interview and gain insight into specific sections.

During the interviews, participants were asked to describe how the T1 and T2 assessments were currently being used, how frequently they thought the assessment should be administered, perceived usefulness of enhancements to web-based displays, and preferences for the structure of self-reported score results. Caregivers and coaches were asked how they would prefer to see the results in figure format. To facilitate discussion about the format of the results, some caregivers and coaches were provided with example figures for discussion.

All interviews were conducted via Zoom with video being disabled. With participants’ permission, the interviews were audio recorded and transcribed. The caregivers were given a US $25 gift card as compensation for their time. During and after the interviews, interviewers took notes describing patients’ responses and impressions using semistructured debriefing forms.

### Ethics Approval

This study was reviewed and exempted by the Duke University Institutional Review Board (protocol number Pro00105250).

### Inclusion Criteria

Adults aged >18 years who completed at least 1 session with a coach in either the RCI REACH or OFC program were invited to participate. Eligible coaches included RCI REACH and OFC coaches who had coached at least 1 RCI-affiliated caregiver.

### Recruitment, Consent, and Sampling

Recruitment was conducted in 2 ways. First, the Duke study team met with the RCI coaches on the web during a regularly scheduled coach meeting to introduce the study. At the meeting, coaches were provided with information about the study, which included an information sheet and a link to a secure web-based questionnaire (via REDCap [Research Electronic Data Capture; Vanderbilt University]), where coaches and caregivers could review written information about the study and answer screening questions. The coaches were asked to share the study information and REDCap link with caregivers. If an individual was eligible after completing the screening, the Duke study staff reached the coach or caregiver to provide more information and schedule the interview.

The participants were also recruited from the contact list provided by the RCI. Duke study team members reached out to potential participants via email and telephone to provide information about the study and subsequently enrolled the individual if they were eligible and expressed interest in participating in the study. Eligibility and screening responses were recorded via REDCap for both strategies.

During the recruitment process, the study team obtained information about the duration of caregiving, type of caregiving (child, spouse, or parent), time participated as a coach in the RCI REACH or OFC programs, and basic sociodemographic information. This information was used to ensure the diversity of experiences in the study sample. Purposive sampling was used to achieve a balanced representation of interviewees from each existing program (OFC and REACH), the duration of caregiving, caregiver type, and time enrolled in the program.

### Analysis

Descriptive statistics were used to summarize the characteristics of the study participants. Qualitative content analysis was used to analyze the participant transcripts. The team used NVivo (version 12 for Windows; QSR International) qualitative data analysis software [31] to apply codes to transcripts, segmenting sections of the transcripts that were associated with particular concepts discussed in the interviews.

The analysts created a codebook (Multimedia Appendix 1) starting with deductive codes created from the interview guides and adding inductive codes identified during the initial review of the debriefing forms. To establish intercoder reliability, 3 analysts (NL, ED, and CS) independently coded 2 transcripts and then met to reconcile them. Discrepancies in coding were resolved through discussion and the codebook was refined. Next, the 3 analysts (NL, ED, and CS) divided the remaining number of transcripts and independently coded them. The analysts met once a week (including TC) throughout the process to check the coding process and refine the code definitions, as necessary. If significant changes to coding definitions or new codes were added, all transcripts were reviewed and recorded based on the latest definitions and codebooks. Once the coding was complete, the 3 analysts (NL, ED, and CS) reviewed the code reports and summarized the findings. The team compared the results of the caregivers and coaches. Structural and qualitative content analyses were used to identify themes and summarize the results.

## Results

### Description of Study Participants

In all, 25 caregivers (RCI REACH: 13/25, 52%; OFC: 12/25, 48%) and 11 coaches (RCI REACH: 5/11, 45%; OFC: 6/11, 55%) were included in the study. Table 1 presents the sample characteristics. Most participants were women; 52% (13/25) of the caregivers and 45% (5/11) of the coaches had graduate degrees.

**Table 1:** Background characteristics of the study participants.

Demographic characteristics		Overall caregivers (n=25)	RCI^a^ REACH^b^ caregivers (n=13)	OFC^c^ caregivers (n=12)	Overall coaches (n=11)	
Age (years), mean; median (minimum-maximum)		55; 53.75 (34-74)	64.31; 65 (44-79)	45.6; 42.5 (24-69)	40.5; 40 (24-66)	
Gender (female), n (%)		22 (88)	10 (77)	12 (100)	11 (100)	
Ethnicity (Hispanic), n (%)		5 (20)	2 (15)	3 (25)	2 (18)	
**Race (check all that apply), n (%)**						
	White	19 (76)	11 (85)	8 (67)	8 (73)	
	Black, Indigenous, or people of color	5 (20)	2 (15)	3 (25)	2 (18)	
	Prefer not to answer	1 (4)	0 (0)	1 (8)	1 (9)	
**Highest education completed, n (%)**						
	Completed high school or some college or university	6 (24)	2 (15)	4 (33)	0 (0)	
	Associate degree, college, or university	6 (24)	3 (23)	3 (25)	6 (55)	
	Graduate school	13 (52)	8 (62)	5 (42)	5 (45)	
**Ability to pay for basics in the past month (food, housing, and heat), n (%)**						
	Very hard or hard	3 (12)	2 (15)	1 (8)	0 (0)	
	Somewhat hard	7 (28)	3 (23)	4 (33)	3 (27)	
	Not very hard	15 (60)	8 (62)	7 (58)	8 (73)	
**Length of enrollment in RCI program (caregiver), n (%)**						N/A^d^
	≤1 month	2 (8)	2 (15)	0 (0)		
	1-3 months	13 (52)	8 (62)	5 (42)		
	3-6 months	5 (20)	1 (8)	4 (33)		
	≥6 months	5 (20)	2 (15)	3 (25)		
**Length of time spent as an RCI coach, n (%)**		N/A	N/A	N/A		
	≤6 months				3 (27)	
	6 months-1 year				4 (36)	
	>1 year				4 (36)	

^a^RCI: Rosalynn Carter Institute for Caregivers.

^b^REACH: Resources for Enhancing Alzheimer's Caregiver Health.

^c^OFC: Operation Family Caregiver.

^d^N/A: not applicable.

Most caregivers cared for their spouses or partners (20/25, 80%) or parents (4/25, 16%). The average length of time for caregiving was 4.1 years for RCI REACH (median 4.5; minimum-maximum 21-9) and 4.8 years for OFC (median 4.5; minimum-maximum 0.7-13). Most caregivers (24/25, 96%) lived with their care recipients. Approximately one-third of the coaches had experience coaching in other programs, either within or outside the RCI (4/11, 36%). The average number of caregivers’ coaches who were coached simultaneously was 11 (median 10; minimum-maximum 3-22).

### Mode of Survey Completion

Coaches often administered the assessments to caregivers verbally and entered caregiver responses into the web-based system. The majority of coaches (8/11, 72% of the coaches) used Zoom to administer the assessment, and 27% (3/11; all OFC coaches) conducted the assessment over telephone. A few coaches indicated that before the COVID-19 pandemic, they administered the assessments in person, with the caregivers. Some coaches had the caregivers fill out the assessment in their presence to mitigate the burden of reading aloud every question while also being able to observe important nonverbal cues from the caregivers.

Overall, most caregivers appreciated taking the first assessment with their coach, whether it was via Zoom or telephone. Several caregivers mentioned that taking the assessment with their coach felt more personal, allowed them to share their caregiving story, and build rapport with their coach. There was agreement that the benefit of taking the first assessment with a coach lies in the caregiver’s ability to share their stories. A coach described the first assessment (T1) as follows:

I think you can just garner a lot from what is being said and things that are not being said at T1, and I think that’s where I focus. That’s how I get to know them, and I don’t know whether you’re going session by session with the information, but I think a couple of things. I think the first session should be more of a get to know you session, and although I do tell them about the program in the first session, I focus more on letting them tell their story because I think that’s how: 1) You build the relationship; but then 2) How you’re able to assess the real need at hand.

Two caregivers preferred to take the assessment on the web by themselves, and 3 coaches suggested that caregivers take the assessment on the web by themselves for at least the second assessment. Most caregivers appreciated taking the assessment with their coach to build rapport, so it is unclear if there is a significant value in having the caregiver fill out T2 with their coach because at that point, rapport would already have been formed.

### Frequency of Survey Administration

The assessments were administered in the first (T1) and last (T2) sessions for the RCI REACH and OFC programs. The coaches and caregivers were asked if more frequent assessments would be useful. There was significant interest among caregivers and coaches of both programs in adding assessments midway through the program. Specifically, approximately 40% of the caregivers felt that it would be helpful to take the assessment again midway through the program, in addition to the current pre-post format. Participants generally agreed that the assessment could be used to evaluate the progress and assess the needs of caregivers. A RCI REACH coach said the following:

I think that it would be helpful to maybe even do it at the halfway point. That may help get some more scores then and to see if something needs to be reassessed or re-evaluated in how we continue the program for the caregiver. That way if there is something else that has come up or if we see a significant change in score, we can reflect that with the caregiver and decide how to proceed with the rest of the program.

The importance of assessing caregivers’ needs frequently was highlighted by 2 RCI REACH participants, who emphasized that their care recipients’ needs would change frequently (weekly or monthly). One major detractor for not wanting to add a midway assessment was how long it would take (ie, coaches and caregivers believed it would add burden). Some caregivers and coaches suggested that the middle assessment should be condensed or completed on the web without the coach.

### How the Assessment Scores Were Used

The caregivers were asked how they and their coaches used their assessment responses. The assessment did not include the functionality for caregivers to see their scores on their own. However, most caregivers remembered going over their assessment results to their coaches. Many caregivers remembered that their coaches provided them with resources after reviewing the assessment results*.* A caregiver said the following:

...We focused a lot on the ones that I was scoring the least on. And she tried to give me pointers and other resources if I wanted to take a look at them on my own.

Of the few caregivers who did not recall the results of the assessment with their coach, there was agreement that this practice would be helpful. A caregiver said the following:

Yeah, but would I have appreciated it or gotten something out of it? Yeah! I answered questions for over an hour, so if somebody had sent me a printout or talked to me about it – ...It would have been helpful to get that feedback about that very first hour.

At the time of data collection, the assessment tool provided score reports only to the coaches, and these score reports were available only at the topic level. Coaches said they use assessment responses to structure encounters, as well as identify where and what caregivers are struggling with so they can provide resources and support. Coaches also confirmed what caregivers speculated: that the baseline assessment was used to initiate dialogue between the coach and caregiver, build rapport, and gain a sense of how the caregiver is doing.

A few coaches expressed dissatisfaction with how they currently see results, namely that formatting is not helpful or user-friendly, and that the results do not provide much useful information about what to do next. A coach said the following:

I think most definitely having more information about what they responded and why it’s a concern, and what would be the proper steps to take would be very beneficial because right now we have, we see that happy face and it gives us an explanation. The answers they gave us are concerning, but then that’s all.

Some coaches had trouble with the assessment tool or did not value it. For example, 2 coaches said that they had to take good notes during the assessment administration because they could not access the results. A RCI REACH coach said that they administered the assessment because it was a requirement but did not use the assessment responses at all; they felt that they could gain the information they needed through conversations with the caregiver during the coaching sessions.

### Facilitating Score Report Review in the Future: Caregivers

Caregivers and coaches were asked what score report features would be most useful in the future to inform coach-caregiver encounters. Caregivers were asked if they were interested in seeing individual questions or topic-level scores from the assessments. Table 2 shows caregiver preferences for viewing scores from the assessments (one REACH participant was not asked this question and another REACH participant indicated that they were not interested in seeing their results on the assessments at all).

**Table 2:** Caregiver preferences for viewing assessment results.

	Topic scores only, n (%)	Individual scores only, n (%)	Both scores, n (%)
RCI^a^ REACH^b^ (n=11)	3 (27)	3 (27)	5 (45)
OFC^c^ (n=12)	4 (33)	2 (17)	6 (50)
Total (N=23)	7 (30)	5 (22)	11 (48)

^a^RCI: Rosalynn Carter Institute for Caregivers.

^b^REACH: Resources for Enhancing Alzheimer's Caregiver Health.

^c^OFC: Operation Family Caregiver.

Of the 7 participants who expressed wanting to see only topic-level scores, 5 (71%) mentioned that they felt that going through the individual item scores would be too burdensome because of the length of the assessment. One caregiver remarked, “I think it’s just a better quick snapshot of where you’re at in things, rather than an overwhelming individual number for every question.”

Of the caregivers who wanted to be able to see both the topic-and individual-level scores, more than half (6/11, 55%) wanted to see topic-level scores first so they could see overall progression, regression, or change over time; then, if certain areas did not change or scores regressed, they wanted to be able to view individual question scores for more details: “After seeing it by topic, if I have questions – then we can dive in for the question for that particular topic.”

Of the 23 caregivers, only 5 (22%) wanted to see individual item scores. One reason for this was that some caregivers wanted to see “all the details” and changes at the individual item level.

Most caregivers were interested in seeing the results and changes in their scores over time, but only a few caregivers were interested in seeing the scores that improved. Two caregivers were not interested in viewing the scores on the assessments that worsened (they only wanted to see scores for the topics or individual questions where they improved). These caregivers said that seeing worsening scores would not make them feel good about their progress. Some caregivers also mentioned that seeing the scores on the assessments would provide extra validation to help them see the program really helping them. When asked if reviewing the scores on the assessment would be helpful, a caregiver responded as follows:

[Yes], because I think it’s a little bit more concrete, a more visual way to understand where you’re at. Gosh, I scored really high or I scored really low or maybe things aren’t as bad as I thought they were...

A few caregivers emphasized the importance of being able to interpret figures showing the results of their scores. For example, some caregivers mentioned that the figures needed to have clear legends to indicate which scores were good or bad or if they were moving in a positive or negative direction.

### Facilitating Score Report Review in the Future: Coaches

Most coaches also wanted to see both topic level and individual item scores (7/11, 64%). In all, 3 coaches did not want to see individual item scores; 2 of them being OFC coaches who felt that going through individual item scores would be too burdensome because of the length of the assessment. Three coaches indicated that they would only want to look at individual item scores for specific high-risk topics such as safety or depression. Coaches were open to multiple formats and felt that sharing results with caregivers would be helpful in showing caregiver progress. A coach noted the following:

...I think it would be really cool for us to show them at the end of the program or even if another period of assessment’s introduced, checking in with them whenever that’s done, just to show them progress and how we’re doing.

Overall, most coaches thought it would be beneficial to share all changes in assessment results with caregivers (scores that improved, worsened, or remained the same). A coach said the following:

And at the end, I just want to know if they’ve improved or if they’ve gone down then I can refer resources or do what I need to do at that point.

However, 2 of the coaches were concerned that some caregivers may be discouraged or more stressed if they were told that their scores worsened or did not improve over the course of the program. One of these coaches said the following:

I think it would depend on the caregiver. I think some would find it stressful that they’re not meeting their goal, but others might find it motivating.

However, overall, both caregivers and coaches felt that seeing and sharing the caregivers’ improvement scores would make them caregiver and coach feel validated. A few coaches were not interested in sharing scores with caregivers; instead, they felt that the dialogue they would have with the caregiver would be more important in explaining how the caregiver has changed from T1 to T2. Two coaches suggested that the scoring be optional to the caregiver or used for goal setting.

Overall, both caregivers and coaches were extremely interested in reviewing scores on assessments throughout the RCI programs, with particular interest in how scores changed over time, and whether caregivers improved in certain areas or still needed support in others. Some wanted to see topic scores for a quick summary, whereas others wanted to delve into the details to see exactly where the changes were happening. Most of the coaches and caregivers were open to either option.

### Comparing Caregivers

Caregivers were asked if they were interested in seeing how their scores on the assessments were compared with other caregivers in their respective programs. Across both programs, most caregivers (approximately two-thirds) said that they were not interested in seeing how they were compared with the other caregivers. One of the main reasons given by caregivers in both programs was that every caregiver is different and in a different situation, so comparing oneself to another was not useful. A caregiver said the following:

I don’t think that [comparing caregivers] would be helpful because...in caregiving everybody’s situation is different, everybody is caring for somebody different, and it’s probably not relevant to you.

The other main reason caregivers were not interested in seeing how they compared with the other caregivers was that they feared that it would cause them stress and anxiety and make them feel worse. A caregiver stated the following:

...*If I had bad scores, then I’d be feeling like, “Okay, why are my scores not as good as theirs? Like, what am I doing wrong as a caregiver?” So, I wouldn’t want to compare.*

Of the caregivers who were interested in seeing how their scores compared with the other caregivers (8/24, 33%), the reasons for this were curiosity and getting a sense of where they stand compared with the other caregivers. Overall, coaches were less certain about whether they would like to compare caregiver scores on the assessments, and opinions varied according to the RCI program. Some OFC coaches noted that it would be helpful to compare caregivers because they could look at average scores and identify any trends or patterns. RCI REACH coaches were less enthusiastic about comparing caregiver scores on assessments. In general, they provided the same reasons as caregivers (ie, every caregiver’s situation is different, and it is not useful to compare one to another). In addition, RCI REACH coaches were concerned that sharing score information with caregivers would cause more stress. A few OFC coaches (2/5, 40%) also shared the concern of adding additional stress, so they would keep this information to themselves and not share it with caregivers. The other 3 OFC coaches believed the opposite (ie, that it would actually be helpful for caregivers to see how they compare with others; it could be a learning experience and help validate their feelings).

## Discussion

### Principal Findings

This study aimed to describe how a web-based assessment is currently being used to identify and monitor caregiver well-being in 2 RCI programs and describe perceived enhancements to the web-based system that could support caregiver-coach encounters by directing priorities for the encounters.

Although the questionnaires included in the assessments were self-administered, RCI coaches used the questionnaires to elicit conversations and get to know the caregivers in their programs. Most assessments were administered verbally via Zoom or telephone, a practice that was likely initiated due to the COVID-19 pandemic but was also well accepted by caregivers and coaches. Coaches valued being able to observe the caregivers on Zoom while administering the questionnaires because they could pick up on nonverbal cues. At least 1 coach indicated that they preferred to share the questionnaire with their caregivers during the assessment.

Historically, the assessments were administered twice: once at the beginning and once at the end of each program. Coaches and caregivers generally agreed that more assessment administrations would be useful because they could receive feedback on caregivers’ status and adjust the program as needed. The frequency of change in the care recipient should be considered when deciding the assessment frequency to pick up on new caregiver needs and have coaches able to address these needs in a timely manner. The primary concern regarding the addition of assessment administrations was the length of the assessment. One potential consideration for further refinement of the assessment is to prioritize topics that need to be specifically evaluated by individual caregivers and administer those topics only. Another way to reduce the time required to complete assessments is for caregivers to complete the follow-up assessments on their own because they have already built rapport with coaches; one risk of this approach is that caregivers may not complete a lengthy follow-up assessment on their own. Coaches could encourage caregivers to complete the assessment before the last meeting with their coaches.

Assessment scores were visible to the coaches but were not accessible to the caregivers. Most caregivers agreed that having access to scores would be useful for viewing their status and identifying areas where they needed the most help from their coaches. Caregivers who do not remember going over the assessment results with their coach may become frustrated with the assessment process if they do not think their coach is looking at the results. A formal portal for caregivers to review their own scores or review their scores in tandem with their coach would allow the process of reviewing scores with coaches to be more memorable and useful.

In general, coaches valued the assessments and indicated that they used the scores to identify areas where caregivers found them difficult. A number of coaches were unhappy with the current functionality of the assessment score results format and emphasized the need for a clear interpretation of the assessment results. Coaches and caregivers provided a wide range of feedback on how they preferred to see assessment scores in the future and what would be most useful to them. Overall, most caregivers and coaches were interested in seeing the score results over time, indicating improvement or worsening. Most caregivers and coaches prefer access to topic- and question-level scores. There was considerable heterogeneity in how caregivers described why they preferred different levels of detail in their score reports. Coaches and caregivers were generally in support of viewing changes in scores in figure format. Some concerns about this practice were mentioned, such as caregivers feeling discouraged about a lack of progress or worsening. This opens an opportunity to reframe the assessment as a tool or intervention to identify needs rather than weaknesses. Most coaches and caregivers were not interested in comparing caregiver scores across other caregivers in the programs, although a few felt that it might be useful. Conflicting perspectives by both caregivers and coaches on whether they would like to compare caregivers to other caregivers demonstrate the importance of providing options for assessment result displays. The heterogeneity in preferences for the format of the results highlights the need for flexibility in the functionality of the assessment tool, with the ability to toggle higher- or more specific-level information, or whether improvement or worsening scores are displayed. One way to address this in the context of the program would be for coaches to ask caregivers about their preferences and adjust the score output for each caregiver’s perspective.

The importance of being able to understand and interpret the assessment result figures was mentioned by a few participants. Building on the work that has been accomplished in the interpretation of figures in clinical care settings [32-35], additional research could evaluate the key features of figures that influence interpretation and understanding. Clear figure and score interpretation is critical for coaches and caregivers to determine which needs should be prioritized.

### Building on This Study

Future steps for refining the assessment could include developing individualized functionality or features for caregivers and coaches. For example, it is possible that some caregiver characteristics, such as caregiver status (eg, caregiver is doing well or finding it difficult) could be associated with preferences for assessment functionality. Future research could investigate these characteristics and use them as predictors for the presentation of assessment functionality to individualize the assessment experience for caregivers. Another opportunity for individualizing assessment functionality is to set goals and track outcomes toward these goals. The goals of the programs were individualized for each caregiver based on the coach-caregiver encounters, and assessment scores were used to track these goals. Assessment score reports may need to be individualized to draw attention to the outcomes that are most important to the caregivers. Clear interpretation of guidelines for scores is also important. For example, the goal of the coaching intervention may be more protective in nature; therefore, caregivers should expect to see relatively consistent scores over time rather than an improvement.

### Strengths

A key strength of this study was that the qualitative interviews included perspectives of the interventionists (coaches) and individuals receiving the interventions (caregivers). Stakeholder interviews are often conducted in clinical care contexts but focus on the individuals receiving interventions: patients. By including both stakeholders in the qualitative study design, we compared the results, resulting in a holistic set of insights and suggestions for the next steps. Another strength is that we had the opportunity to interview caregivers who were currently enrolled in RCI programs, as well as caregivers who had completed their RCI program. The results of this qualitative study will drive the next steps for RCI’s web-based platform and expand on current standards for administering self-reported questionnaires in clinical practice settings. The results of this study could potentially be useful for health organizations when building or upgrading web-based portals for patients and clinicians.

### Limitations

The results of this study should be considered in light of its limitations. First, the interview participants were only from RCI coaching programs; the generalizability of the functionality of the tool may be limited. Second, all coaches included in the interviews actively participated in the RCI programs; therefore, their interviews may reflect a more positive outlook than coaches who were no longer with RCI. Finally, the caregivers and coaches who dropped out of the program were not included in the study; consequently, the results may reflect a more positive view of the assessment.

### Conclusions

We conducted qualitative interviews with the coaches and caregivers in the 2 RCI programs. Web-based and procedural enhancements were identified to enrich caregiver-coach encounters. New and enhanced strategies for using web-based assessments to direct priorities in the caregiver-coach encounters included (1) integrating figures showing caregiver progress at the individual caregiver level, (2) ability to toggle results through different figures focused on individual versus aggregate results, and (3) support for interpreting scores. The results of this qualitative study will drive the next steps for RCI’s web-based platform and expand on current standards for administering self-reported questionnaires in clinical practice settings.

## Appendix

Multimedia Appendix 1 The final codebook used to code the qualitative transcripts.

## Abbreviations

OFC: Operation Family Caregiver
PRO: patient-reported outcome
RCI: Rosalynn Carter Institute for Caregivers
REACH: Resources for Enhancing Alzheimer's Caregiver Health
REDCap: Research Electronic Data Capture
