# Supplementary material for: Prioritizing Support Offered to Caregivers by Examining the Status Quo and Opportunities for Enhancement When Using Web-Based Self-reported Health Questionnaires: Descriptive Qualitative Study
Source: JMIR Form Res. 2022 Apr 8;6(4):e30877. doi: 10.2196/30877 (PMC9034415; doi:10.2196/30877)
Supplement: Multimedia Appendix 1 [file formative_v6i4e30877_app1.docx]

| Codes | Description |
| --- | --- |
| Background Information | Apply to descriptions of why coaches and caregivers joined REACH/OFC (what attracted them). Include caregiver experiences as a caregiver. |
| Goals | Apply to descriptions of what coaches and caregivers hope to gain by the time the program is complete |
| Success | Apply to text describing how caregivers and coaches define "success", including how caregivers' definitions of success have changed over time. |
| Prioritizing Support | Apply to text describing how coaches and caregivers decide/prioritize support during the encounters. |
| Quitting | Apply to text describing if caregivers ever considered quitting the program or not. |
| Assessment Responses | Apply to descriptions of how caregivers and coaches use the responses on the assessment to identify areas where the caregiver needs support, including how coaches use them to structure the encounters, and how caregivers perceived the assessment was used. |
| Remove | Apply to text describing what topics and/or questions on the assessment could be removed. Also include any text describing if coaches/caregivers want certain aspects of the assessment to be changed or modified, including condensing topics, changes to response options, if questions are confusing, things they don't like about the assessment, etc. |
| Does not apply personally | Apply to text describing what assessment topics and/or questions did not apply to caregiver or coaches think don’t apply to a lot of their caregivers |
| Mode of Completion | Apply to text describing how caregivers and coaches complete the assessment. Include what caregivers and coaches like and dislike about conducting the assessment via that mode. |
| Not Covered | Apply to text describing what topics are not covered by the assessment that caregivers are receiving support on, or that coaches or caregivers think the assessment should cover. Include if caregivers are not receiving support on other topics not covered by assessment. |
| Important Topics | Apply to text describing what topics coaches think are the most important topics to track on the assessment and why |
| Frequency | Apply to descriptions of how frequently coaches and caregivers think the assessment should be completed to evaluate progress in the program and why. |
| Topic Scores | Apply to text describing when caregivers and coaches discuss seeing assessment scores at the topic level and why (if caregivers also talk about seeing individual item scores in the same paragraph and it's ambiguous, include that as well) |
| Individual Scores | Apply to text describing when caregivers and coaches discuss assessment scores at the individual question level and why (if caregivers also talk about seeing topic level scores in the same paragraph and it's ambiguous, include that as well) |
| Changes over time | Apply to descriptions of how caregivers and coaches would like to see how the assessment results change over time. Include if they want to see changes for individual questions, for certain topics, and if they want to see improvement, worsening or results that stayed the same. |
| Comparing Caregivers | Apply to text where caregivers describe if they do or don't want to see how their assessment results compare to other caregivers. Include if coaches think it would be helpful to compare caregiver scores on the assessment. |
| Formatting Results | Apply to text describing what format (graph, table, scale) caregivers and coaches want to see results in. |
| Level | Apply to text describing if coaches think caregivers and coaches should see the same information or level of detail in regards to assessment scores. Also include if coaches think caregivers should see results from the assessment at all. |
| Under Representation | Apply to text describing any other important aspects of the caregiver-coach encounter or caregiver assessment that we did not ask about, but that's important to know |
| Hypothetical | Use when person is providing an answer that does not apply to them but they think may be helpful for others. |
| Length of Assessment | Apply to any text describing how caregivers/coaches feel about the length of the assessment |
| Feelings and Emotions | Apply to any text describing how caregivers and coaches feel about the program (what they like about the program, what they don't like, etc.). Do not include what they don’t like about the assessment (should go under "remove" code) |
| COVID-19 | Apply to any text describing how COVID-19 has impacted the caregiver/coach and their participation in the program |
